# A Review on Platensimycin: A Selective FabF Inhibitor

**DOI:** 10.1155/2016/9706753

**Published:** 2016-01-28

**Authors:** Manik Das, Partha Sakha Ghosh, Kuntal Manna

**Affiliations:** Department of Pharmacy, Tripura University (A Central University), Suryamaninagar, Tripura 799022, India

## Abstract

Emerging resistance to existing antibiotics is an inevitable matter of concern in the treatment of bacterial infection. Naturally occurring unique class of natural antibiotic, platensimycin, a secondary metabolite from* Streptomyces platensis*, is an excellent breakthrough in recent antibiotic research with unique structural pattern and significant antibacterial activity. *β*-Ketoacyl-(acyl-carrier-protein (ACP)) synthase (FabF) whose Gram-positive bacteria need to biosynthesize cell membranes is the target of inhibition of platensimycin. So, isolation, retrosynthetic analysis, synthesis of platensimycin, and analogues of platensimycin synthesized till today are the objectives of this review which may be helpful to further investigate and to reveal untouched area on this molecule and to obtain a potential antibacterial lead with enhanced significant antibacterial activity.

## 1. Introduction

The majority of the “illness” lies in the fact that when immune system is defeated, it is in war with pathogens. Development starting from ethnic to modern synthetic approaches in drug discovery mainly provides better weapons to combat and survive against pathogens. In contrast to medical development, pathogens also have acquired protection called “resistance.” More or less all classes of antibiotics are resistant to bacteria; hence a novel process to the discovery of antibiotic with new mechanism of action is essential [1, 2]. Microbial and chemical groups at Merck in the year 2006 have found three novel chemical classes, using antisense technology, from their older microbial screening library [3]. Two compounds, platensimycin and platencin, were found as potent inhibitors of fatty acid biosynthesis. Platensimycin selectively inhibits fatty acid acyl carrier protein synthase II (FabF). Platensin is a balanced dual inhibitor of both FabF and FabH (fatty acid acyl carrier protein synthase III) [4]. The third compound lucensimycin A was found to inhibit ribosomal protein synthesis. FabF is one of the enzymes which catalyze the biosynthesis of fatty acids in bacteria. It makes FabF an essential target for inhibiting bacterial growth in resistant bacteria. Previously two classes of inhibitor, cerulenin [5] and thiolactomycin [6–8], were reported, but inhibitory activity was poor (IC_50_ ranges within 1.3–13 *μ*g/mL) with poor antimicrobial activity (*Streptococcus aurous*, MIC—64 *μ*g/mL) [9]. On the other hand, platensimycin had shown selective FabF inhibitory activity on* S. aurous* and* E. coli* with IC_50_ value of 48 nM and 68 nM [3]. The ability of this class of compounds to bind and inhibit FabF enzyme has given a new class of antibiotics. Among them platensimycin and platencin are most promising and need further investigation. There might be an argument that if fatty acid synthesis is an attractive target for antibacterials, why there have not many drugs or natural inhibitors targeting this pathway been isolated? One of the reasons may be that the* Streptomyces* and related* Actinomyces*, the organisms that have delivered most of the existing antibiotics, are constrained in their ability to produce fatty acid synthesis inhibitors by the adjacent relationship between the synthetic pathways of fatty acids and polyketides. These pathways share many chemical, mechanistic, and structural features, and, thus, fatty acid synthesis inhibitors recurrently inhibit polyketide synthesis (e.g., cerulenin is a powerful inhibitor of polyketide synthesis). An organism producing a fatty acid synthesis inhibitor must retain not only a resistant form of the fatty acid synthetic enzyme but also a resistant form of the antibiotic-producing polyketide synthase. Furthermore, if several polyketides are needed for survival of the organism in its ecological niche, then resistant forms of each of these pathways would be prerequisite. Such precincts seem likely to severely narrow the opportunities to evolve fatty acid synthesis inhibitors and may account for the scarceness of such antibiotics. However, various* Streptomyces* produce analogues of thiolactomycin, which has not been reported to block polyketide synthesis, thus demonstrating that development of fatty acid synthesis inhibitors by this organism is conceivable [10].

## 2. Isolation of Platensimycin

Platensimycin was isolated by Wang et al. in 2006 at concentration of 2 to 4 mg/L from fermentation broth of* S. platensis* (MA7327 and MA7331) using SephadexLH20 liquid chromatography medium by reversed-phase HPLC chromatography [3]. In a subsequent study, three-step isolation was modified by Singh et al. and they established two-step method eliminating the SephadexLH20 step [11]. It was also isolated from* Streptomyces platensis* (MA7327), recovered from soil samples collected in Eastern Cape, South Africa [12].

## 3. Structure of Platensimycin

**Figure 1:**
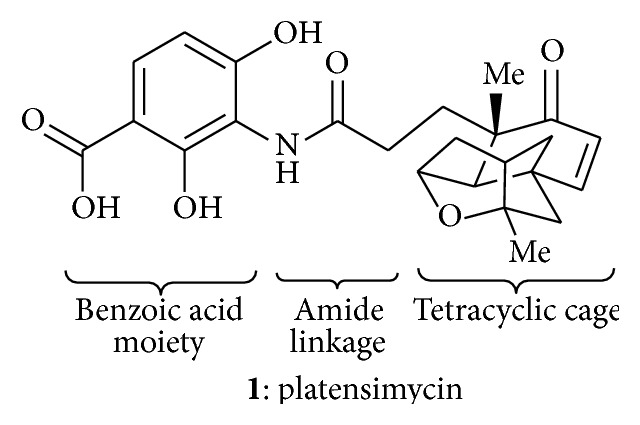
Structure of platensimycin.

Platensimycin (Figure 1) consists of a benzoic acid moiety substituted at ortho and para with hydroxyl group and in meta position is conjugated with a unique pentacyclic ketolide by an amide linkage [13]. The structure was established by combination of DQ-COSY and TOCSY correlations (2D NMR), UV, and IR spectroscopy and confirmed by X-ray crystallography [14].

## 4. Synthesis of Platensimycin

Platensimycin consists of an aromatic acid conjugated with aliphatic moiety by an amide linkage. The effective synthetic strategy is to synthesize the aromatic and aliphatic parts separately and then combine them by amide linkage. Many synthetic methods are available for the synthesis of those two building blocks.

### 4.1. First Total Synthesis of Platensimycin

Nicolaou et al. in the year 2006 first reported the total synthesis of platensimycin [26].

#### 4.1.1. Retrosynthetic Analysis of Platensimycin

**Scheme 1:**
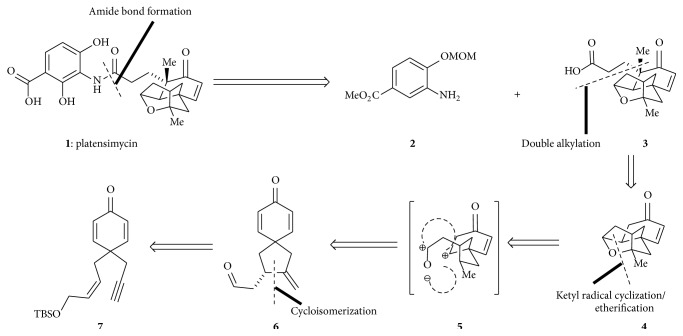
Retrosynthetic analysis of platensimycin.

Using retrosynthetic analysis (Scheme 1) they separated the aromatic amine** 2** and the carboxylic acid** 3** by disconnection of amide linkage. The carboxylic acid was further simplified to a simplified enyne in successive three retrosynthetic steps. This resulted in two target molecules to synthesize the tetracyclic carboxylic acid and the aromatic amine from simplified starting material.

#### 4.1.2. Synthesis of Tetracyclic Cage

**Scheme 2:**
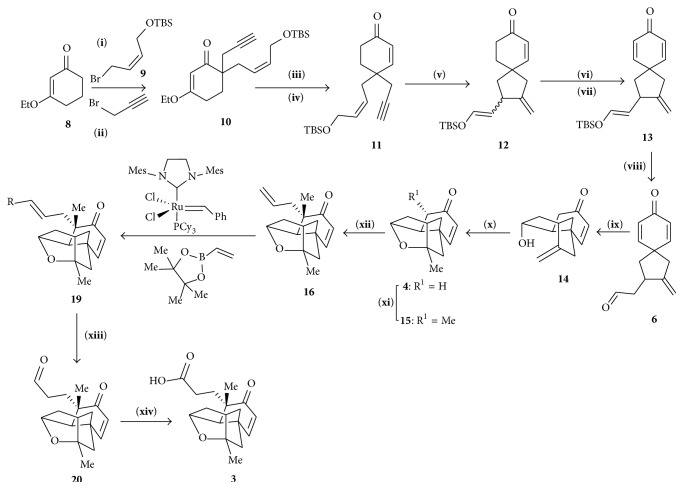
Synthesis of tetracyclic cage. (**i**) LDA, (**ii**) LDA, (**iii**) DIBAL-H, then HCl, (**iv**) TBSCL, (**v**) [CpRu(MeCN)_3_]PF_6_, (**vi**) LiHMDS, TMSCl, (**vii**) Pd(OAc)_2_, (**viii**) HCl aq., (**ix**) Sml_2_, HFlP, (**x**) TFA, (**xi**) KHMDS, MeI, (**xii**) KHMDS, (**xiii**) Me_3_NO, and (**xiv**) NaClO_2_.

The simplified enone generated from 3-ethoxycyclohex-2-enone which was used as a starting material (Scheme 2). Allylic bromide** 9** [27] (LDA, 92%) and propargyl bromide (LDA, 97%) were used as reagents of choice to generate the bis-alkylated enone** 10** from** 8**. Reduction followed by acidic hydrolysis and reintroduction of the TBS ether produced enone** 11** from enone** 10** (yield 84%). Spirocycle** 12** was generated by cycloisomerization of** 11** [28, 29]. Oxidation of** 12** produced bis-enone** 13** [30] which upon acid hydrolysis gave desired aldehyde** 6**. Secondary alcohol** 14** was prepared by addition of samarium(II) iodide in a dilute solution of aldehyde** 6** HFIP in THF/HMPA followed by NH_4_Cl solution. Esterification of** 14** with TEA resulted in the formation of cage-like structure** 4** which on treatment with KHMDS and MeI followed by KHMDS and allyl iodide produced olefin** 16**. Vinyl pinacol boronate and** 16** reacted in presence of the Grubbs second generation catalyst to produce vinyl boronate** 19** which on reacting with trimethylamine N-oxide gave** 20**. Following Pinnick protocol** 20** was converted to desired carboxylic acid** 3**.

#### 4.1.3. Synthesis of Aromatic Amine

**Scheme 3:**
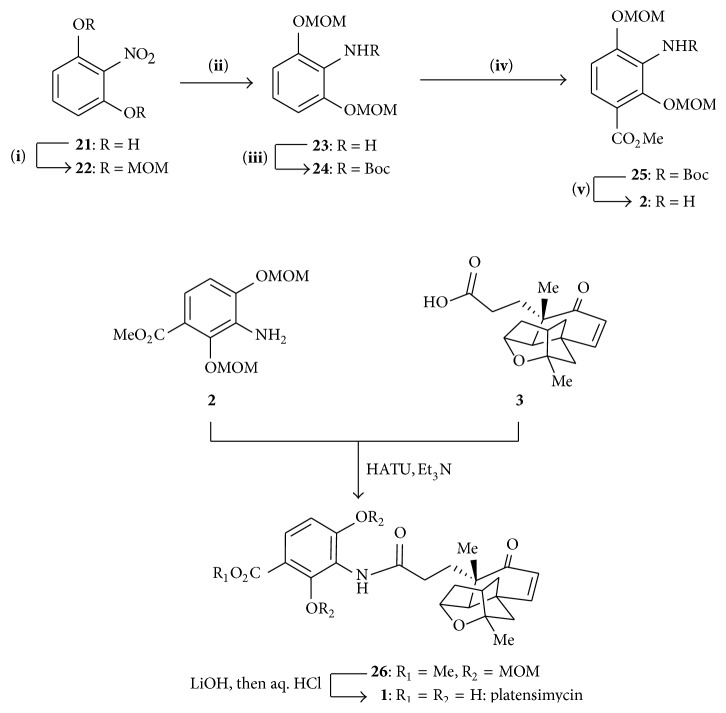
Synthesis of aromatic amine and platensimycin. (**i**) NaH, MOMCL, (**ii**) H_2_, Pd/C cat., (**iii**) Boc_2_O, (**iv**) nBuLi, and (**v**) 205°C.

The synthesis of the aromatic amine** 2** was started from 2-nitroresorcinol** 21** by protecting with MOM ether, followed by catalytic hydrogenation,** 24** was formed (Scheme 3). Again protecting the amino group and followed by silylation, lithiation, and quenching with methyl cyanoformate** 24** was carboxylated and by unprotecting amino group using thermolysis, desired aniline** 2** was synthesized.

#### 4.1.4. Synthesis of Platensimycin Core

The total synthesis of platensimycin was completed by the coupling of carboxylic acid** 3** with aniline** 2** which was achieved by treatment with HATU followed by hydrolysis with LiOH (Scheme 3).

### 4.2. Another Approach for Synthesizing Tetracyclic Cage by Nicolaou et al. 

After successfully reporting the first total synthetic strategy of platensimycin, Nicolaou et al. [31] report a new synthetic strategy that starts from the readily available and inexpensive (R)-(−)-carvone to the tetracyclic enone** 2**.

#### 4.2.1. Retrosynthetic Strategy of Tetracyclic Enone

**Scheme 4:**
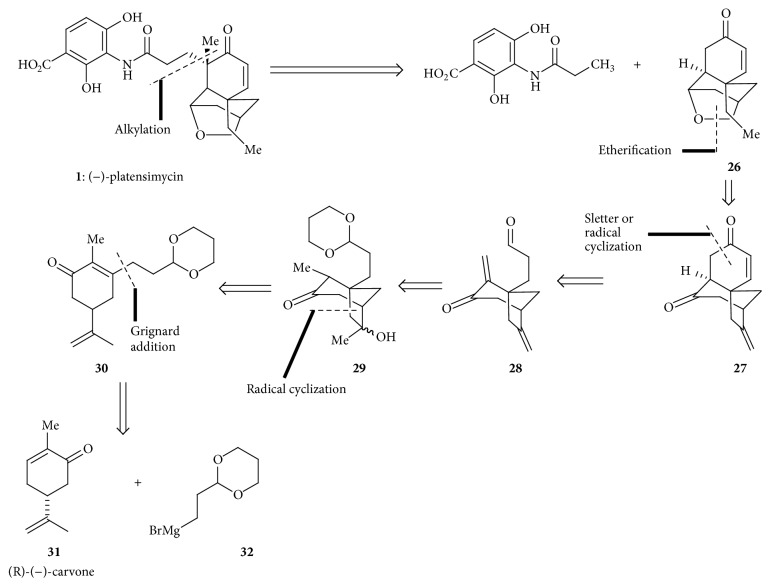
Retrosynthetic analysis of platensimycin.

A five-step retrosynthetic disconnection approach was shown to generate commercially available (R)-(−)-carvone** 31** from** 26** (Scheme 4).

#### 4.2.2. Synthesis of Tetracyclic Enone

**Scheme 5:**
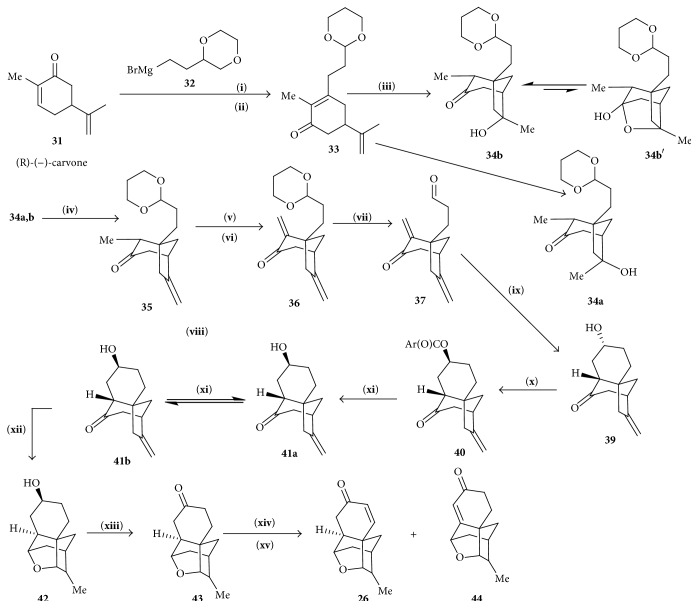
Synthesis of tetracyclic cage. (**i**) CeCl_3_, (**ii**) PCC, and (**iii**) Hg(OAc)_2_, then NaBH_4_, (**iv**) Martin's sulfurane, (**v**) TMSCl, Lil HMDS, and then PhSeCl, (**vi**) H_2_O_2_, Py, (**vii**) AcOH, MW, (**ix**) Sml_2_, (**x**) DIAD, ArCO_2_H, PPh_3_, Ar=p-NO_2_C_6_H_4_, (**xi**) KOH, (**xii**) L-selectride (H_3_O+), (**xiii**) PCC, (**xiv**) TMSCl, Lil HMDS, and (**xv**) IBX or Pd(OAc)_2_.

(R)-(−)-carvone** 31** on reaction with Grignard's reagent** 32** using CeCl_3_ [32] and by oxidizing with PCC gave the corresponding enone** 33**. Enone** 33** was treated with Hg(OAc)_2_ and later on with NaBH_4_ to generate approximately 1 : 1 mixture of exo- and endotertiary alcohol, which on dehydration using Martin's sulfurane reagent produced exocyclic alkene** 35** which was treated with TMSCl and HMDS and followed by an electrophilic quench with PhSeCl and subsequent oxidative elimination (H_2_O_2_) to give enone** 36**. Enone** 37** was treated with SmI_2_ for radical cyclization, and by adding mentioned reagent (Scheme 5) in subsequent 8 steps of tetracyclic cage** 26** was synthesized.

### 4.3. Matsuo's Synthesis of Tetracyclic Cage

#### 4.3.1. Retrosynthetic Analysis of Tetracyclic Cage

**Scheme 6:**
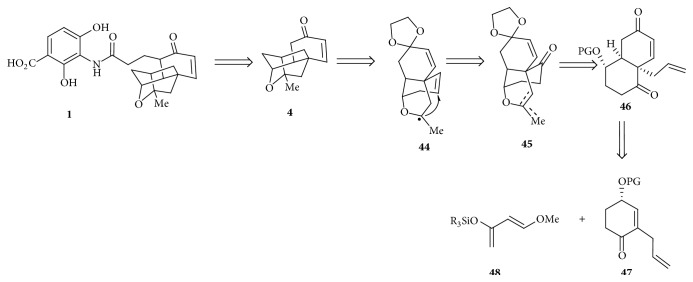
Retrosynthetic analysis of platensimycin.

Matsuo et al. [33] offered another route for stereocontrolled synthesis of tetracyclic enone** 4**. They expected that the transannular radical cyclization of** 44** will produce enone** 4**. A four-step retrosynthetic analysis is producing enone** 47** and siloxydiene** 48**. The key feature of plan is that all stereocenters in** 4** are controlled by the stereochemistry presented in** 47** (Scheme 6).

#### 4.3.2. Synthesis of Tetracyclic Cage

**Scheme 7:**
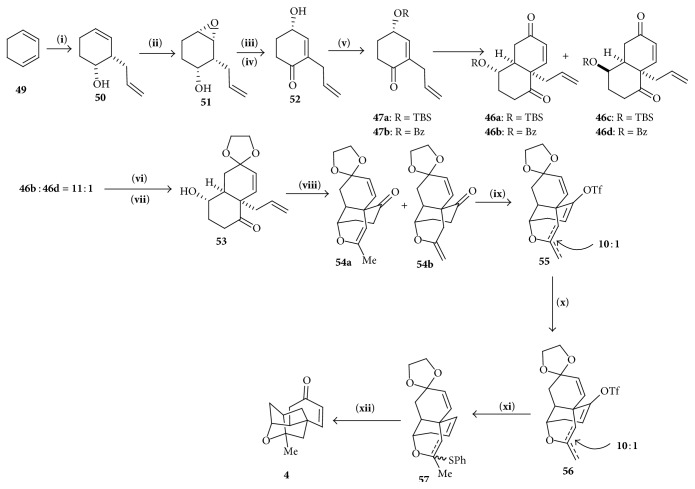
Synthesis of tetracyclic cage. (**i**) allylBBr_2_, then HOOH NaOH, (**ii**) t-BuOOH, VO(sese)_2_, (**iii**) Dess-Martin, periodinane, (**iv**) Silica gel, (**v**) TBSCl imidazole/BzCl, Py, (**vi**) PMSOTf (cat.) (TMSOCH_2_)_2_, (**vii**) aqNaOH separation of diastereomers, (**viii**) PdCl_2_ Cu(OAc)_2_ DMA, O_2_, (**ix**) KHMDS, (**x**) Pd(OAc)_2_(PPh_3_)_2_ HCOOH, Bu_3_N, (**xi**) PhSH, (**xii**) Bu_3_SnH AIBN toluene, reflux, and (**xiii**) 1N aq HCL.

*O*-TBS and* O*-benzoyl-protected enones** 47a** and** 47b** were prepared from 1,3-cyclohexadiene** 49** through allylboration, selective oxidation, and utilizing the Dess-Martin oxidation, with the yield of (97%). Diels-Alder reaction in between** 47a** and** 47b** and siloxydienes produced two stereogenic mixtures** 46a**,** 46b** and** 46c**,** 46d**. The mixture of two inseparable diastereomers (**46b** and** 46d**) was employed in the next step. Following Noyori's procedure [26] followed by hydrolysis of the benzoyl group and separation of diastereomers** 53** was given which upon catalytic oxidation using palladium(II) chloride and copper(II) acetate produced** 54a** and** 54b** in 10 : 1 ratio. Vinyl triflate** 55** was produced from** 54** and reduced to** 56** which was transformed to** 57**. Transannular radical cyclization of monothioacetal** 57** gave desired** 4** (Scheme 7).

### 4.4. Kaliappan's Synthesis of Tetracyclic Cage

#### 4.4.1. Retrosynthetic Analysis of Tetracyclic Cage

**Scheme 8:**
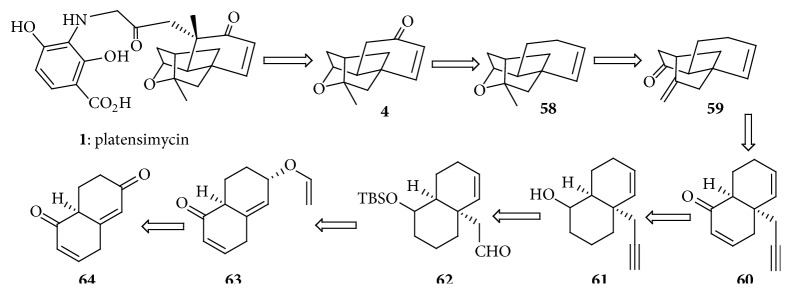
Retrosynthetic analysis of platensimycin.

Kaliappan and Ravikumar [34] showed seven-step retrosynthetic analysis using a 5-exo-trig radical cyclization strategy [35, 36] to generate Wieland-Miescher ketone** 64** (Scheme 8).

#### 4.4.2. Synthesis of Tetracyclic Cage

**Scheme 9:**
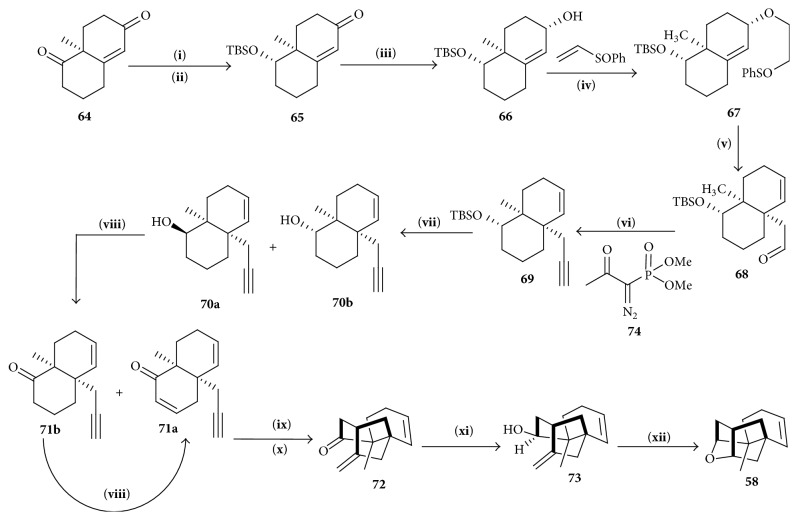
Synthesis of tetracyclic cage. (**i**) NaBH_4_, ETOH, 00C, 2 h, (**ii**) TBSCI, Im, DMF, r.t., 2 h, (**iii**) DIBAL-H CH_2_Cl_2_, (**iv**) NaH, cat. KHTHF, r.t., 12 h, (**v**) Decalin, 180°C, 5d, (**vi**) K_2_CO_3_, MeOH, (**vii**) TBAF, THF, reflux, (**viii**) IBX PhF/DMSO 65°C, (**ix**) TBTH, AIBN t-BuOH, reflux, 12 h, (**x**) PPTS, CH_2_Cl_2_, r.t., 6 h, (**xi**) L-selectride, THF, −78°C to r.t., and (**xii**) TFA/CH_2_Cl_2_, 0°C, 2 h.

Synthesis was started from Wieland-Miescher ketone** 64** [37], a chiral starting material, which on two-step reduction yielded an inseparable diastereomeric mixture of alcohols** 66** [38]. Corresponding aldehyde** 68** containing oxoethylene group was synthesized from alcohols** 66** by Mandai protocol [39]. Using Ohira-Bestmann reagent** 74** [40, 41], enyne** 69** was formed which was treated with AIBN in* tert*-BuOH and PPTS to initiate the radical cyclization resulting formation of tetracyclic cage** 72**. L-Selectride, THF, and TFA/CH_2_Cl_2_ were consecutively added with** 72** to produce the desired enone** 58** (Scheme 9).

### 4.5. Corey's Synthetic Strategy

#### 4.5.1. Synthesis of Tetracyclic Cage

**Scheme 10:**
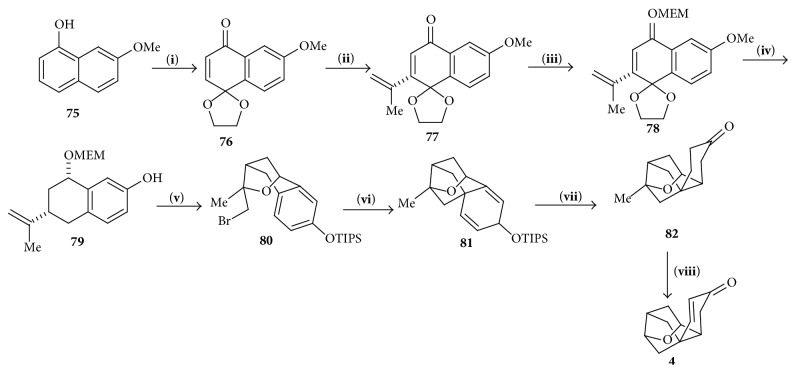
Synthesis of tetracyclic cage. (**i**) PhI(O_2_CCF_3_)_2_, OHCH_2_CH_2_OH, MeCN, 0°C, 2 h. (**ii**) (*S*)-BINAP, [Rh(cod)_2_]BF_4_, C_7_H_8_, H_2_O, Et_3_N, CH_3_-CH(BF_3_K)=CH_2_. (**iii**) (1) NaBH_4_, MeOH, (2) MEMCl, *i*-Pr_2_NEt, CH_2_Cl_2_, (3) TsOH, Me_2_CO, 0°C, (4)* i*-Bu_2_AIH, CH_2_Cl_2_, 0°C, (5) Et_3_SiH, (CF_3_CO)_2_O, CH_2_Cl_2_, −20°C. (**iv**) PhSH, Cs_2_CO_3_, DMF, D 170°. (**v**) (1)* i*-Pr_3_SiCl, imidazole, CH_2_Cl_2_, 23°C, 12 h and (2) Br_2_, CH_2_Cl_2_, −78°C. (**vi**)* n*-Bu_4_NF, THF, D 130°C. (**vii**) [Rh(cod)_2_]BF_4_, (*R,R*)-DIOP, H_2_ 600 psi, CH_2_Cl_2_, 16 h. (**viii**) (1) TMSOTf, Me_3_N, CH_2_Cl_2_ and (2) IBX, MPO, DMSO.

Lalic and Corey [42] used methoxy *α*-naphthol** 75** as the starting material which upon reaction with bistrifluoroacetoxyiodobenzene and ethylene glycol in acetonitrile at 0°C produced 6-methoxy-1,4-naphthoquinone-4-ethylene ketal** 76**. An enantioselective conjugation of 2-propenyl group to ketal** 76** using 2-propenyl trifluoroborate, Rh-BINAP, BF_4_ catalyst, and triethylamine [43–45] produced the chiral ketone** 77**.* cis*-tetralin** 78** was generated from** 77** by reduction of the carbonyl group, hydroxyl protection, and reductive cleavage of the ethylene ketal subunit. Demethylation of** 78** produced phenol** 79** which was converted to tricyclic** 80** by etherification followed by reaction with Br_2_ in CH_2_Cl_2_. Heating with tetra-*n*-butylammonium fluoride in THF at 130°C, tetracyclic cage** 81** was produced which on catalytic diastereoselective hydrogenation produced tetrahydro derivative** 82**. The saturated ketone** 82** was transformed into the corresponding *α*,*β*-enone** 4** using the 2-iodoxybenzoic acid sequence (Scheme 10) [46, 47].

### 4.6. Yamamoto's Synthetic Strategy

#### 4.6.1. Retrosynthetic Analysis of Tetracyclic Core

**Scheme 11:**
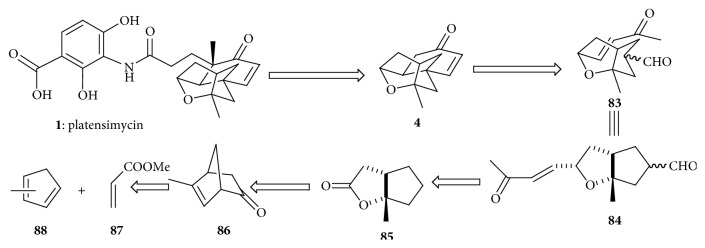
Retrosynthetic analysis of platensimycin.

Li et al. [47] used an intramolecular Robinson annulation approach [48] in the retrosynthetic analysis presented in (Scheme 11). The bicyclic compound** 84** to give the tetracyclic core structure** 4** is key step in the retrosynthesis by Robinson annulation event.

#### 4.6.2. Synthesis of Tetracyclic Core

**Scheme 12:**
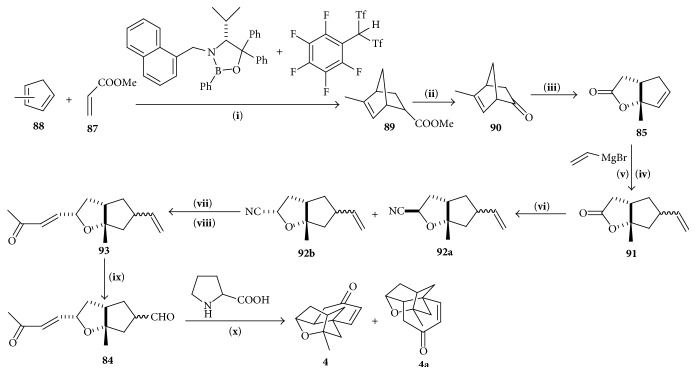
Synthesis of tetracyclic cage. (**i**) CH_2_Cl_2_, −78°C, 14 h, (**ii**) LDA, THF, −78°C, then PhNO, −78°C, 2 h, and then LiOH dioxane/H_2_O 30°C, 20 h, (**iii**) H_2_O_2_/NaOH Et_2_O/H_2_O 0°C to r.t., 45 min, (**iv**) CuBr·Me_2_S, CH_2_CMgBr THF/Me_2_S, −40°C to r.t., (**v**) 4.5 mol% HNTf_2_, CH_2_ClCH_2_Cl, 70°C, 45 min, (**vi**) DIBAL-H, toluene, −78°C, 30 min, then Et_2_AlCN BF_3_·OET_2_, 20 min, (**vii**) DIBAL-H/*n*-BuLi, −78°C to 0°C, 25 min, (**viii**) NaH, CH_3_COCH_2_P(O)(OET)_2_, THF, 0°C, 20 min, (**ix**) 2.02 eq NaIO_4_, 3.5 mol% RuCl_3_ 6 : 1 CH_3_CN/H_2_O r.t., 3 h, and (**x**) 1 eq pyrrolidine-2-carboxylic acid, DMF, r.t., 5 days, then 2N NaOH(aq) 0°C to r.t., 40 min.

The bicyclic ketone** 84** can be synthesized from known lactone** 85** which could be generated from ketone** 86**, through a Baeyer-Villiger oxidation/rearrangement sequence [49, 50] and by utilizing Bronsted acid assisted chiral Lewis acid catalyzed Diels-Alder reaction, and subsequent N-nitrosoaldol addition/decarboxylation, ketone** 86** could be easily prepared from inexpensive, commercially available starting materials (Scheme 12).

Diels-Alder reaction between methyl acrylate** 87** and methyl cyclopentadiene** 88** produced adduct** 89**. The reaction was catalyzed by BLA and carbon-based Bronsted acid [51]. Ketone** 90** was obtained in one pot reaction from adduct** 89** using lithium enolate and lithium hydroxide in dioxane. Baeyer-Villiger oxidation in basic condition [52] of ketone** 90** gave lactone** 85**. Vinyl lactone** 91** was obtained from lactone** 85** using cuprate reagent [53] and trifluoromethanesulfonimide [54, 55]. DIBAL-H reduction followed by cyanation produced cyanide** 92a**,** 92b**, which was reduced and subjected to Wadsworth-Emmons conditions [56] to give enone** 93**; ruthenium-catalyzed oxidation [57] produced aldehyde** 84**. Using L-proline as the chiral control element followed by sodium hydroxide treatment gave the desired tetracyclic core structure** 4**.

## 5. Pharmacology of Platensimycin

**Figure 2:**
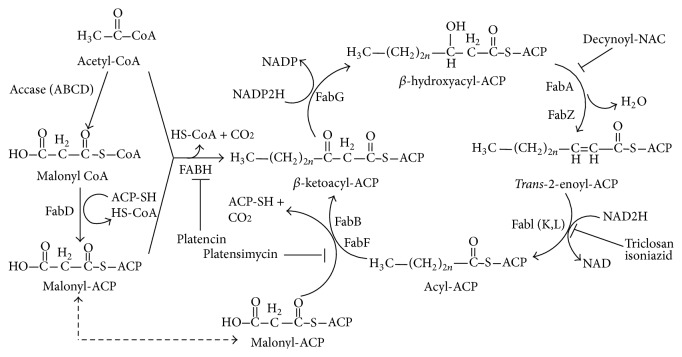
Fatty acid biosynthesis pathway of bacteria (FAS II).

Bacterial cell wall synthesis, protein synthesis, and DNA replication are the predominant targets for widely used antibiotics. But the emergence of resistance to antibiotics demands new antibacterial targets. Fatty acid synthase (FAS) pathway is now an attractive target for antibacterial agents because, as a new target, FAS inhibition will not suffer from bacterial resistance immediately and biosynthesis pathway of bacteria, plants, and parasites (FAS II in which component proteins are dissociated) is different from mammals (FAS I in which component proteins are generally single-chain, multidomain homodimers or two-chain heterodimers carrying all proteins of the pathway) in subcellular organization of components which demonstrate a target specificity for the FAS II inhibitors. A general scheme for type II fatty acid biosynthesis is shown in Figure 2[9]. A recent development in finding inhibitors of fatty acid biosynthesis is the discovery of platensimycin which shows broad-spectrum Gram-positive antibacterial activity (*Staphylococcus aureus* (MRSA) and* Enterococci* (VRE), MIC < 1.0 *μ*g mL^−1^) by selectively inhibiting cellular lipid biosynthesis [58]. The mechanism of action is the selective inhibition of elongation of FabF (a condensing enzyme) in the bacterial fatty acid synthetic pathway by intercalating with the malonyl binding site of the catalytic triad of FabF acyl enzyme intermediate. Inverse correlation of FabF expression levels with the sensitivity of* S*.* aureus* to the drug platensimycin confirms that FabF is the useful target for antibacterial action. Platensimycin exhibited an IC_50_ of 48 nM and 160 nM against FabF in* S*.* aureus* and* E*.* coli*, respectively. But the drug shows weak inhibition against FabH with an IC_50_ value of 67 mM. Further studies have shown that* in vitro* binding of platensimycin with FabF is relatively weak, which leads to the discovery of its binding with acyl-thioester intermediate of the FabF pathway. From a crystal structure of a Cys-163-Gln FabF mutant, which simulates acyl-thioester intermediate, it was found that platensimycin bind with the active site of FabF with the carboxylic acid group lying in the malonate-binding site coplanar with the amide side chain of Gln163 [59].

Though Brinster et al. [60] explained an alternative hypothesis that FAS II inhibition is not a suitable target for* Streptococcus agalactiae* (lactobacillales). The need for synthesized fatty acid by their own (streptococci, pneumococci, enterococci, and staphylococci) reduces in the presence of exogenous fatty acid in both* in vitro* and* in vivo* conditions. Human serum has a high composition of fatty acids, so FAS II inhibitor may not affect at all Gram-positive pathogens* in vivo*. But the findings with* S. agalactiae* can be reasonably extended to all Gram-positive bacteria, which has started a vigorous debate. Also there is no clear explanation how bacteria incorporates exogenous fatty acids into their cell [61, 62]. Platensimycin exhibited antibacterial activity against efflux-negative* Escherichia coli* (*tolC*), but not against wild-type* E. coli*, specifying that efflux mechanisms, and not compound specificity, limit the effectiveness of platensimycin in* E. coli* and possibly other Gram-negative bacteria [3].

## 6. Platensimycin Analogues

The major drawback to most natural products, including platensimycin, is poor pharmacokinetic properties and negligible oral bioavailability. Only the continuous infusion of a high platensimycin dose showed effectiveness in mice infected with* S. aureus* [3]. For the improvement of pharmacokinetic profile, many researchers have developed platensimycin analogues (Table 1); however potent antimicrobial activity than that of platensimycin is yet to get.

## 7. Conclusion

Development of bacterial resistance to the existing antibiotics is an alarming situation in the 20th century. Finding new target for bacterial demolition is essential. Platensimycin serving as a potential antibiotic interacting with FabF may bypass bacterial resistance. It should be noted that starting from 2006 till now a huge number of scientists provided many synthetic strategies and derivatives which are very encouraging. Though some evidence like incorporation of exogenous fatty acid inside bacteria when supplied makes the hope regarding platensimycin is uncertain, overall, isolation of platensimycin as a selective FabF inhibitor, complex synthesis of tetracyclic cage, and enhancement of its pharmacokinetic properties by its derivative synthesis are the excellent works in the era of medicinal chemistry. It is expected that this review might be helpful for the medicinal chemist.

## Figures and Tables

**Table 1:** Antimicrobial activity of synthesized platensimycin analogue.

Sl. number	Analogues name	Analogues structure	Antimicrobial activity	Reference
1	(−)-Platencin	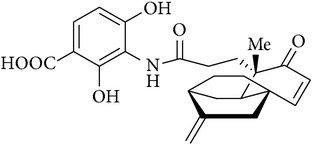	Inhibits both FabF and FabH with similar potency	[12]

2	7-Phenylplatensimycin	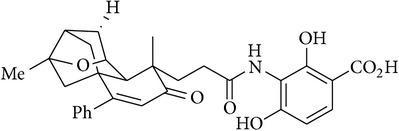	MIC against *S. aureus* (MSSA), 0.25 *μ*g/mL	[15]

3	11-Methyl-7-phenylplatensimycin	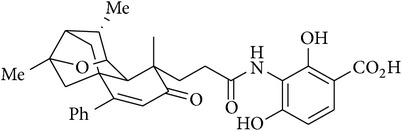	MIC against *S. aureus* (MSSA), <0.25 *μ*g/mL	[15]

4	Sulfonamide analogues of platensimycin	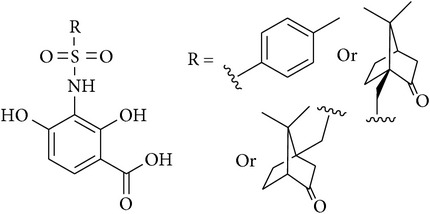	Not tested	[16]

5	Oxazinidinyl platensimycin	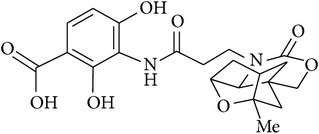	MIC of 90 *μ*g/mL against *S. aureus*, *S. agalactiae*, and *B. subtilis*	[17]

6	Isoplatencin	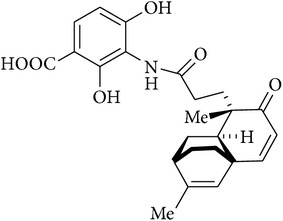	MIC against *S. aureus* (MSSA), 0.4 *μ*g/mL	[18]

7	Cl-*iso*-platencin	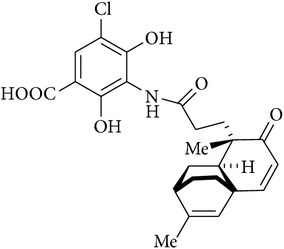	MIC against *S. aureus* (MSSA), >25.6 *μ*g/mL	[18]

8	Cl-platencin	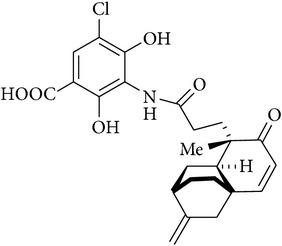	MIC against *S. aureus* (MSSA), >25.6 *μ*g/mL	[18]

9	Dehydrohomoplatencin	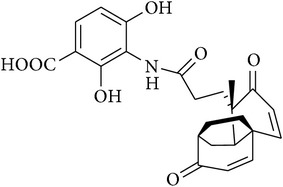	MIC against *S. aureus* (MSSA), 0.4 *μ*g/mL	[19]

10	Isoplatensimycin	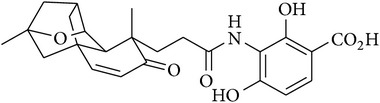	MIC against *S. aureus* (MSSA), 128 *μ*g/mL	[20]

11	Carbaplatensimycin	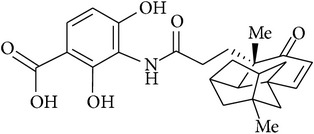	MIC against *S. aureus*, 0.4–1.1 *μ*g/mL	[21]

12	Platensimycin B_1_ and B_3_	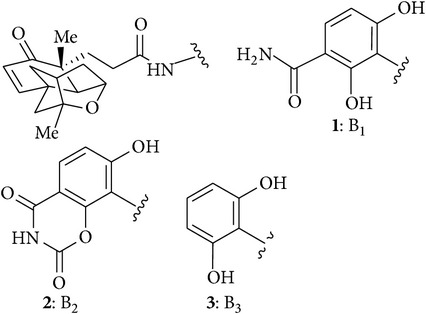	Not tested	[22]

13	Platensimide A	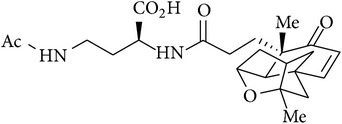	Not tested	[22]

14	Homoplatensimide A and homoplatensimide A methyl ester	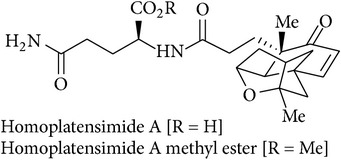	Not tested	[22]

15	Dialkylamino-2,4-dihydroxybenzoic acids analogues of Platensimycin	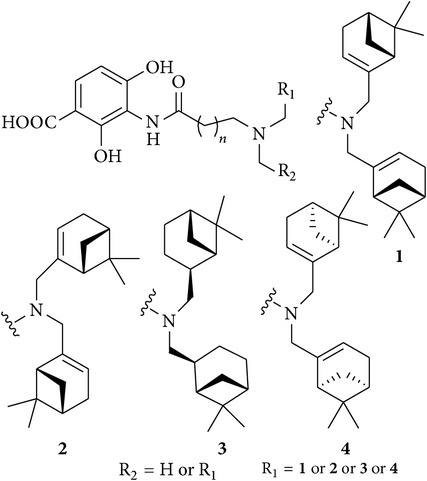	Out of 18 Synthesized derivatives, four derivatives which have the substitution 1, 2, 3, and 4, respectively, have shown potential activity against *B*. *subtilis* (MIC 2–8 *μ*g/mL)	[23]

16	(−)-nor-platencin	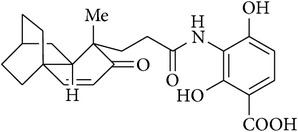	MIC against *S. aureus* (MSSA), 5 *μ*g/mL	[24]

17	Adamantaplatensimycin	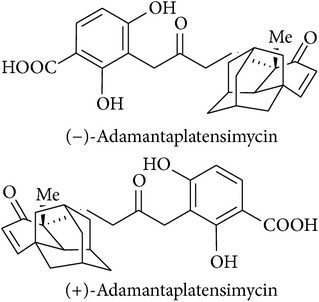	MIC of (−)-adamantaplatensimycin against *S. aureus* (MSSA), 1.3–1.8 *μ*g/mL; MIC of (+)-adamantaplatensimycin against *S. aureus* (MSSA), >88 *μ*g/mL	[25]

## Abbreviations

Ac: Acetyl
AIBN: 2,2′-Azobis(isobutyronitrile)
BINAP: 2,2′-Bis(diphenylphosphino)-1,10-binaphthalene
Boc: * tert*-Butoxycarbonyl
Bz: Benzoyl
DIBALH: Diisobutylaluminium hydride
DMF: N,N,-Dimethylformamide
DMSO: Dimethyl sulfoxide
HATU: O-(7-Azabenzotriazol-1-yl)-N,N,N′,N′-tetramethyluronium hexafluorophosphate
HMPA: Hexamethyl phosphoramide
KHMDS: Potassium hexamethyldisilazide
LDA: Lithium diisopropylamide
PCC: Pyridinium chlorochromate Piv pivaloyl
PPTS: Pyridinium-*p*-toluenesulfonate
Py: Pyridine
TEA: Triethylamine
Tf: Trifluoromethanesulfonyl
TFA: Trifluoroacetic acid
TFE: 2,2,2-Trifluoroethanol
THF: Tetrahydrofuran
TMS: Trimethylsilyl.
